# Multi-Instance Classification of Breast Tumor Ultrasound Images Using Convolutional Neural Networks and Transfer Learning

**DOI:** 10.3390/bioengineering10121419

**Published:** 2023-12-13

**Authors:** Alexandru Ciobotaru, Maria Aurora Bota, Dan Ioan Goța, Liviu Cristian Miclea

**Affiliations:** 1 Department of Automation, Faculty of Automation and Computer Science, Technical University of Cluj-Napoca, 400114 Cluj-Napoca, Romania; ciobotaru_alex@yahoo.com (A.C.); dan.gota@aut.utcluj.ro (D.I.G.); 2 Department of Advanced Computing Sciences, Faculty of Sciences and Engineering, Maastricht University, 6229 EN Maastricht, The Netherlands; botaaurora62@gmail.com

**Keywords:** breast cancer, ultrasound images, Convolutional Neural Networks, deep learning, transfer learning, computer vision

## Abstract

Background: Breast cancer is arguably one of the leading causes of death among women around the world. The automation of the early detection process and classification of breast masses has been a prominent focus for researchers in the past decade. The utilization of ultrasound imaging is prevalent in the diagnostic evaluation of breast cancer, with its predictive accuracy being dependent on the expertise of the specialist. Therefore, there is an urgent need to create fast and reliable ultrasound image detection algorithms to address this issue. Methods: This paper aims to compare the efficiency of six state-of-the-art, fine-tuned deep learning models that can classify breast tissue from ultrasound images into three classes: benign, malignant, and normal, using transfer learning. Additionally, the architecture of a custom model is introduced and trained from the ground up on a public dataset containing 780 images, which was further augmented to 3900 and 7800 images, respectively. What is more, the custom model is further validated on another private dataset containing 163 ultrasound images divided into two classes: benign and malignant. The pre-trained architectures used in this work are ResNet-50, Inception-V3, Inception-ResNet-V2, MobileNet-V2, VGG-16, and DenseNet-121. The performance evaluation metrics that are used in this study are as follows: Precision, Recall, F1-Score and Specificity. Results: The experimental results show that the models trained on the augmented dataset with 7800 images obtained the best performance on the test set, having 94.95 ± 0.64%, 97.69 ± 0.52%, 97.69 ± 0.13%, 97.77 ± 0.29%, 95.07 ± 0.41%, 98.11 ± 0.10%, and 96.75 ± 0.26% accuracy for the ResNet-50, MobileNet-V2, InceptionResNet-V2, VGG-16, Inception-V3, DenseNet-121, and our model, respectively. Conclusion: Our proposed model obtains competitive results, outperforming some state-of-the-art models in terms of accuracy and training time.

## 1. Introduction

Breast cancer (BC) is arguably one of the primary causes of mortality among women worldwide [1]. Over the past three decades, the increases in its occurrence and fatality rates have been related to the fact that this type of cancer is particularly silent when it comes to its evolution [2]. The abnormal multiplication of cells in breast tissue leads to the creation of a lump known as a tumor, which might be benign or malignant. Usually, most people discover their condition through regular screening. Others may present with an unintentionally found breast lump, a change in breast form or size, or nipple discharge.

**Figure 1:**
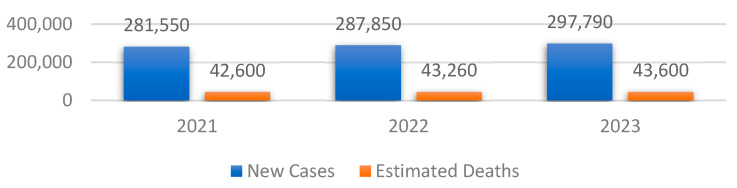
Overview of the total number of BC cases and their associated estimated deaths between 2021 and 2023 in the United States.

In order to emphasize the impact of BC on women in the United States, three studies were selected from 2021 to 2023, respectively [3,4,5]. Figure 1 shows the values of the new cases as well as the estimated deaths of BC among women.

One can note that both the total number of new cases and the associated estimated deaths have increased since 2021, with 16,240 and 1000, respectively. Therefore, the early detection of the symptoms that lead to this type of cancer is crucial to reduce the number of new cases in the following years.

Ultrasound is a frequently used tool in diagnosing BC. Despite its cost-effectiveness and non-invasive nature, this method presents challenges in terms of interpretation and diagnosis. Due to the high volume of material, the efficacy of the method is heavily reliant on the expertise, knowledge, and physical condition of the radiologist at the moment of reading [6]. In order to overcome this issue, computer-aided diagnosis systems in conjunction with Deep Learning (DL) techniques have been developed. The purpose of these systems is to enhance the reliability of the diagnostic process, provide accurate interpretations of the medical data, and minimize the errors that the radiologists may make. Even though the DL models have proved their efficiency in computer vision, a set of challenges arise when applying them in the area of BC detection and classification [7]. Firstly, when working with medical data, especially ultrasound images, there is a lack of available materials due to GDPR regulations. Therefore, data augmentation techniques must be used in order to synthetically create data. Secondly, ultrasound machines might introduce electrical noise, which might negatively impact the quality of predictions. Another significant challenge lies in the realm of unsupervised learning for the annotation of BC ultrasound images. While supervised learning-based models have demonstrated improved outcomes, obtaining annotated breast images from expert clinicians is exceedingly challenging in practical settings. The majority of the breast ultrasound images that are accessible lack proper labeling. Lastly, most researchers have utilized clinical datasets, which are private datasets. Hence, it is difficult to make meaningful comparisons of the performance of these models in various research studies. This paper addresses, in a comparative way, the problem of Transfer Learning (TL) applied to Breast Ultrasound (BUS) tumor detection and classification using some state-of-the-art DL architectures such as ResNet-50, MobileNet-V2, InceptionResNet-V2, VGG-16, Inception-V3, or DenseNet-121. Each state-of-the-art model is fine-tuned using a custom Deep Neural Network (DNN) architecture, which will also be explained in detail. Additionally, a simple model architecture composed of a Convolutional Neural Network (CNN) and DNN is also proposed and compared with the rest of the models to see if it can achieve or outperform them. What is more, the issue regarding the lack of data is also addressed by developing an image augmentation algorithm using geometric transforms.

The following is the structure of the paper: Section 2 presents the related work that was undertaken in the field of BUS image classification using both DL techniques. Section 3 shows the materials and procedures that were used to detect and classify the type of tumor present in the ultrasound image. Section 4 presents the experimental results that were obtained after training the models. Finally, Section 5 shows the discussions regarding the previously presented experiments and Section 6 presents the conclusions and further research directions.

## 2. Related Work

The area of BC detection and classification has been extensively discussed by the researchers; therefore, various approaches exist in the literature. Therefore, several comprehensive literature reviews were published from 2020 to 2021 [8,9,10]. Regarding this matter, this section highlights related work in this area.

The first selected study was undertaken by Hanan Aljuaid et al. [11] and focuses on BC classification by combining DNNs and TL on the BreakHis [12] dataset. Additionally, they address both the binary and multi-class classification of benign and malignant breast tissues, as well as four subtypes of benign/malignant tissues, using the following models: ResNet-18, ShuffleNet, and Inception-V3. What is more, data augmentation techniques such as random reflection or translations along the *x* or *y* axis were also used to avoid overfitting and help the model generalize better. The results that were obtained for the binary classification between malignant and benign masses are as follows: 99.7%, 97.66%, and 96.94% accuracy for ResNet, Inception-V3, and ShuffleNet, respectively. For the multi-class classification, the values of accuracy were the following: 97.81%, 96.07%, and 95.79% for ResNet, Inception-V3Net, and ShuffleNet, respectively. Even though the models differentiate between different types of benign or malignant breast tissues, the authors do not mention the capability of detecting healthy breast tissue. Additionally, they could also include other models in the study such as IncetionResNet-V2 or VGG. The second study [13] compares the Xception, DenseNet-201, InceptionResNet-V2, VGG-19, and ResNet-152 models on the BreakHis dataset. The issue addressed by the authors was a multi-class classification between eight sub-types of malignant and benign breast tissues, as well as the binary classification between them. The best performance was obtained by the Xception model, with accuracy values ranging from 90.22% up to 98.99%. Regarding the data augmentation techniques, the authors only use rotation and horizontal flip. Additional techniques such as contrast or brightness alterations could also be used.

In the study conducted by Aastha Joshi et al. [14], the focus is on the utilization of TL techniques for the early detection of breast tumors in ultrasound images. The dataset utilized in this research is an online version of the BUS Images dataset, which comprises a total of 693 ultrasound images specifically collected for the purpose of classifying breast masses into benign, malignant, and normal classes. This study conducts feature extraction and fine-tuning on the MobileNet-V2 and Inception-V3 models. This study employs traditional TL techniques for various architectures and aims to reduce the number of parameters in the network to gain a deeper understanding of the network’s actual performance. In this particular scenario, the Inception-V3 model demonstrates the highest level, achieving an accuracy rate of 83.84%, while the MobileNet-V2 obtained 82.54% accuracy. While the authors use the accuracy metric to measure the performance of the models, other metrics such as sensitivity and specificity could be included to examine whether the models make correct predictions, or whether they predict false positives (FP) or false negatives (FN). Rakesh Chandra Joshi et al.’s study [15] contains a broad variety of model assessment approaches, intriguing models, and a multi-level evaluation strategy. Two datasets are used in this study, namely the BUS Images dataset as well as a dataset obtained upon request that contains ultrasound images categorized into two classes of BC, benign and malignant. For the TL process, the work uses VGG-19 and YOLO-V3 models and a model implemented by the authors entitled BUS-CNN. Data augmentation gives the model 9430 images to train with. The techniques used for data augmentation include rotations and contrast modifications. The BUS-CNN model is composed of five convolutional layers and five max pooling layers, the activation function that was used being ReLU. The loss function was categorical cross-entropy and the optimizer was stochastic gradient descent. The results that were obtained by the BUS-CNN model in terms of accuracy, sensitivity, and specificity were 96.31%, 92.63%, and 96.71%, respectively.

Uysal et al. [16] propose a multi-class categorization technique for breast ultrasonography samples using, again, the BUS Images dataset. The study targets the comparison between ResNet50, ResNeXt, and 7Chapter 3’sc to assess their efficacy. This work studies VGG-16, a CNN architecture, and augments data using standard approaches. Three nodes were added to VGG-16’s last three levels. The ResNet50 architecture added two nodes, while the ResNeXt model added three. Therefore, the study’s research evaluated the accuracy, f1-score, and area under the curve. ResNeXt outperformed the other models with 85.83% accuracy. However, the models could also be tested on other similar datasets to further validate their robustness.

The work presented in [17] focuses on identifying BC using thermography, DL architectures, and ML techniques. More specifically, the authors use the U-NET model on a dataset containing 170 infrared images [18]. What is more, the segmentation results from the U-NET model were also combined with the Support Vector Machines algorithm in order to generate the final predictions. The researchers obtained an accuracy value of 94.4%, and precision, recall, and F1-Score values of 96.2%, 86.7%, and 91.2%, respectively. Another metric could also be utilized; this was specificity, to see how many false negative predictions were made over the total number of actual FN predictions.

The study conducted by Abeer Saber et al. [19] focuses on applying TL to BC early detection using state-of-the art models such as Inception V3, ResNet50, VGG-19, VGG-16, and Inception-V2 ResNet on the MIAS dataset. They achieved an accuracy, sensitivity, specificity, precision, F1-score, and AUC of 98.96%, 97.83%, 99.13%, 97.35%, 97.66%, and 0.95, respectively. A summarized overview of the previously presented papers is shown in Table 1. Additionally, the corresponding datasets that were used to conduct the experiments from the previously presented papers are shown in Table 2.

The studies presented in Table 1 focus on both binary and multi-class classifications of BC using TL. Regarding the accuracy value, the best result was obtained by Hanan Aljuaid [11], with over 97.8% using the ResNet model.

## 3. Materials and Methods

This section aims to present the materials and procedures that were employed in order to detect and classify the BC from ultrasound images, that is, the data preprocessing steps, data augmentation algorithm, and the architecture of the DL models.

### 3.1. Data Preprocessing

**Figure 2:**
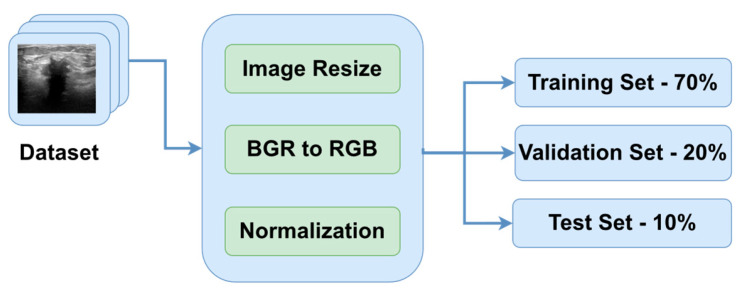
Data preprocessing pipeline.

The dataset employed in this study is entitled the BUS Images dataset. It was collected in 2018 from a group of 600 female patients with ages ranging between 25 and 75 years old [20,21]. The occurrence of BC is highly impacted by the age group of the patient. For instance, the risk of developing in situ or invasive BC is low for women between 10 and 25 years old, as well as for women older than 75 years old. Additionally, women between 40 and 55 years old are predisposed to invasive BC, at 37.3% [22] The prevalence of BC rises markedly with advancing age, reaching its peak after menopause and subsequently declining gradually or remaining stable [23]. Having a collection of ultrasound images collected from patients between 25 and 75 years old is beneficial because it covers the most targeted age range of patients. The dataset comprises a total of 780 BUS images, with the average size of an image being 500 × 500 pixels. There are a total of 437 PNG images representing benign breast tissue, 218 images representing malignant breast tissue, and 133 images representing normal or healthy breast tissue. Mathematically, the dataset is a set of labeled images ${\left\{\left(X_{i}Y_{i}\right)\right\}}_{i=1}^{N}$, where vector X contains the pixel values of the ultrasound images, vector Y contains the target variables, which is one of the previously mentioned categories encoded as strings, and N is the total number of images. The general overview of the preprocessing steps is presented in Figure 2.

**Figure 3:**
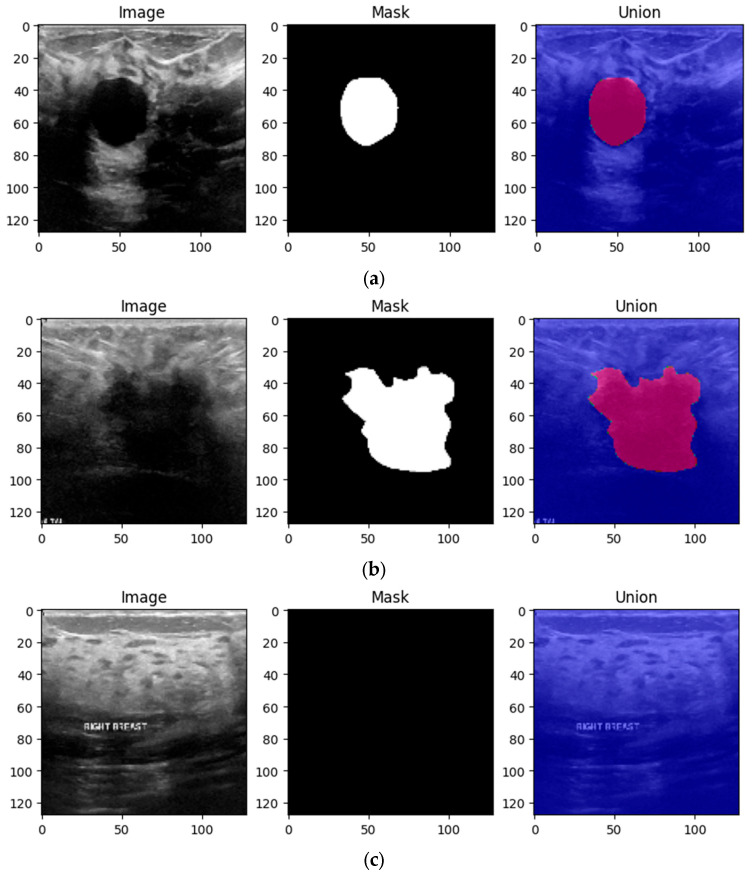
Three preprocessed images from the BUS Images dataset highlighting the ground truth as well as the mask of the three types of breast tissues: (**a**) benign, (**b**) malignant and (**c**) normal.

One of the primary goals of data preprocessing is to improve the accuracy of image data. This is achieved through various techniques such as filtering, speckle noise removal, and contrast enhancement [24,25]. The first step in the preprocessing pipeline is to resize the images in order to make sure that all of them have the same dimensions. Therefore, the images were downsized to 128 × 128 pixels. In this way, the neural networks will be able to better generalize the solution. The next two steps are to convert the channel of the images from BGR to RGB to be compatible with the DL models and normalize the pixel values in the range of [0, 1] by dividing them by 255.0. In order to obtain an extensive understanding of the specific region that the algorithm intended to focus on, Figure 3 is provided, which includes the original image, the ground truth mask, and a colormap representation of the tumor.

The initial form of the dataset only contains 780 images. Therefore, if the model is trained using only these images, it is most likely to overfit and achieve low performance on the test data. Therefore, it is imperative to implement a data augmentation algorithm to generate additional data using geometric transforms. The data augmentation algorithm is presented in Algorithm 1 below. The algorithm requires two inputs: the initial form of the dataset and the multiplication factor. The role of the multiplication factor is to determine how many times the dataset will be augmented. The output of the algorithm is the augmented dataset, which contains both the images and labels as ordered pairs. Then, for each index in the interval [1, multiplication_factor], the corresponding image is augmented using contrast, brightness, flipping, and Gaussian Blur effects. The Adjust_Contrast method accepts two parameters: the ith image in the dataset, which has the batch size, height, width, channels dimsensions, and a contrast factor, which can take both positive and negative values, with the value of 2 being chosen by trial and error.
**Algorithm 1:** Data Augmentation
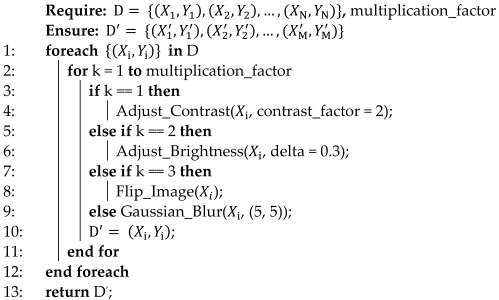


The contrast of each image is computed independently for each channel according to Equation (1), where *X_mean_* represents the mean of the pixels in the image and x represents the component of the pixel along the *x* axis.

(1) $$channel=x-X_{mean}\;\cdot \;contrast\_factor+X_{mean}$$

The Adjust_Brightness method also accepts the ith image in the dataset and another factor, delta. This method transforms RGB images to float representation, changes their brightness, and then returns them to their original data type. Delta represents a floating-point number that may take values in the (−1, +1) interval, and its value is added to each pixel in the image.

**Figure 4:**
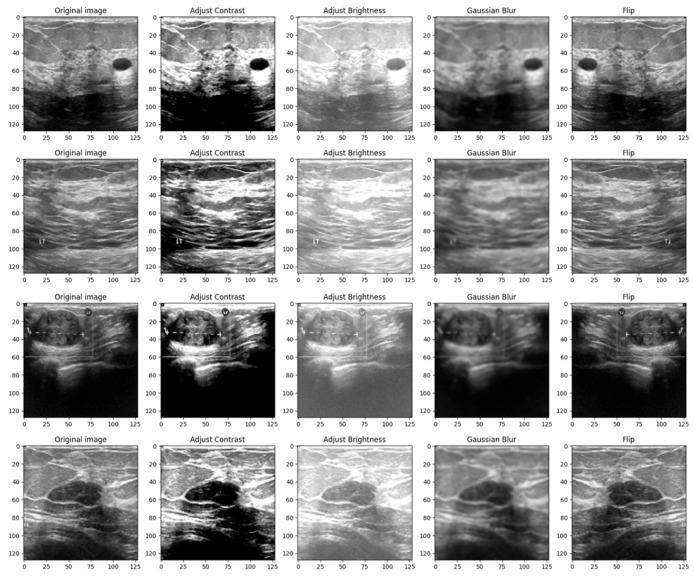
Grid of 4 × 5 random showing the effect of data augmentation on the images from the training set.

Images, like any other signal, might contain many sorts of noise owing to the source, for instance, the ultrasound camera. Therefore, image smoothing techniques such as Gaussian filters help reduce the noise from images taken in low light, smooth the edges, and help the model to generalize the solution efficiently. The kernel used to apply the filter was chosen to be a 5 × 5 matrix [26]. Figure 4 shows a 4 × 5 grid of random BUS images from the training set, highlighting the augmented samples as well as the original ones.

The reasoning behind the decision to alter the contrast, brightness, Gaussian blur and flip was to adapt the model to scenarios in which the tissue masses are not clear enough or need manual adjustments. Even though most ultrasound machines are preset to specific contrast and brightness values for detecting malignant lesions, sometimes the radiologist might need to make these adjustments manually to make sure that the diagnostic is accurate. Moreover, hypoechoic breast lesions are indicative of potential malignancy. On ultrasound imaging, these lesions typically appear darker than the surrounding isoechoic fat [27]. Therefore, in order to train a robust model, contrast and brightness variations should be included. Gaussian Blur was introduced to adapt the model to certain perturbations during screening, such as patient movement. Finally, flip was introduced in order to vary the location of the malignant/benign tissue masses. 

The last step of the algorithm is to split the dataset into three parts to make sure that the model is not tested on the data that was used during the training. Therefore, the train–validation–test split that we chose was 70% training data, 20% validation data and 10% test data [28]. In this study, we compared the results obtained by the models after training them on the dataset augmented with multiplication factors of 5 and 10, respectively. Therefore, if the multiplication factor was equal to 5, the model was trained, validated, and tested on 2730, 780 and 390 BUS images, respectively.

If the multiplication factor was equal to 10, the model was trained, validated, and tested on 5460, 1560 and 780 BUS images, respectively.

### 3.2. Model Fine-Tuning and Training Using TL

TL methods aim to mitigate the challenge of limited data availability and enhance the efficacy of a learner in a specific domain by harnessing knowledge from a correlated domain [29]. The CNN models that were selected to classify the type of BC from the ultrasound images are represented by the following: ResNet50, MobileNetV2, InceptionResNetV2, InceptionV3, VGG16, and DenseNet-121.

In 2014, researchers from Oxford’s Visual Geometry Group (VGG) introduced VGGNet, a novel network architecture. The most frequent VGG variants are those with 16 (VGG-16) and 19 (VGG-19) layers. In our study, we conducted experiments using the VGG-16 version [30], which includes 138 million parameters distributed among 13 convolutional and 3 Fully Connected (FC) layers. The convolutional layers employ a 3 × 3 kernel with a stride of 1 to facilitate the meticulous extraction of features. In addition, the architectural design incorporates pooling layers that are implemented using a 2 × 2 kernel and a stride of 2. The FC layers consist of 4096 units each and are responsible for mapping the features to their corresponding class probabilities in the classification layers. The VGG-16 pre-trained model is frequently utilized in image segmentation, detection, and classification applications. In the same year, 2014, a new parameter-efficient architecture known as GoogleNet or InceptionV1 was released. Even though there are various Inception designs, the most frequent is the InceptionV3 [31] architecture, which was proposed a year later, in 2015, and with which we explored in our research. The InceptionV3 design improved accuracy and decreased the computational complexity of the previous version by leveraging factorization, for instance, by replacing one 5 × 5 convolution with two 3 × 3 convolutions.

InceptionResNetV2 [32] is a DNN architecture that integrates the ResNet and Inception architectures. The utilization of the Inception module and residual networks is integrated into the framework. Additionally, auxiliary classifiers are incorporated to offer supplementary supervision. The multi-scale feature extractor is a computational algorithm designed to extract features from data at multiple scales.

Residual Network (ResNet) is a novel architecture introduced in 2015. ResNet-50 [33,34] is a popular version of ResNet, which includes 50 layers and more than 25 million parameters. This architecture aims to solve the vanishing gradient problem by introducing skip connections that have the capacity to bypass several layers and make a comparison between the input and the desired output. The layers are distributed as follows: 49 convolutional layers, along with 1 global average pooling layer and 1 FC layer. The first layer consists of 64 kernels, with each kernel having dimensions of 7 by 7. The last convolutional layer comprises 512 filters, each with dimensions of 1 by 1.

Google launched MobileNetV1 in 2017, a new mobile-friendly architecture that includes the “Depthwise Separable Convolution” block to minimize complexity and the model size. It is made up of a 33 depthwise convolutional layer that filters the input, followed by an 11-point convolutional layer that combines these filtered values to produce new features by retaining the same number of channels or doubling them. After a year, in 2018, an updated version of the MobileNet architecture was introduced; this is called MobileNetV2 [35], and adds an extra 1 × 1 pointwise CONV layer, which is also called the “Projection layer”, and makes the number of channels smaller, thus making this version of the architecture much smaller in size and faster than the previous one. MobileNetV2 employs an inverted residual structure module (rather than narrow/bottleneck layers in between wide layers of a convolutional block, MobileNetV2 employs wide layers in between narrow/bottleneck layers in a convolutional block, resulting in fewer parameters), an expansion factor “t” (e.g., if the input has 32 channels and the expansion factor t is 6, the internal output will be 32t = 326 = 192 channels), and two types of blocks (one with a stride of 1 and another one with a stride of 2 for downsizing).

DenseNet-121 [36] is a DNN architecture that has, as its core concept, the principle of skip connections, resulting in a robust model. Additionally, it addresses the issue of the vanishing gradient problem and facilitates the propagation of features throughout the model. The architecture comprises compact blocks, intermediary layers for smooth transitions, and bottleneck layers.

**Figure 5:**
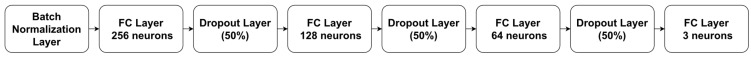
The structure of the DNN that was used to fine-tune the pretrained models.

To optimize the advantages of the current feature extraction capabilities of the architectures and enhance the precision of the classification task, the weights of the state-of-the-art models are frozen while solely training the new layers during the entire procedure. Furthermore, the input layer of the architecture is initialized with the dimensions of the image tensor, which, in this case, are (128, 128, 3). The last layer of each state-of-the-art model is disposed. The learning rate that was used while training the CNN and DNN was 10^−3^. The remaining layers perform feature selection by reducing the dimensionality of the gathered features, as well as feature classification by determining the means to differentiate among the desired output classes. The transformation of the pre-trained model’s 3D output into a 1D feature vector is achieved by employing a DNN composed of a series of Batch Normalization, Dropout and FC layers. The structure of the DNN is presented in Figure 5.

The first layer in the DNN is a Batch Normalization [37] layer. The role of this layer is to provide resistance to the vanishing gradient problem and reduce the training time. Next, three pairs of convolution–dropout layers are added to the structure of the DNN. The first FC layer contains 256 neurons, the second FC layer contains 128 neurons, and the last FC layer has 64 neurons. The role of the Dropout layer [38] is to randomly set to zero a certain number of neurons from its corresponding FC layer each epoch to increase the reliability of the DNN and prevent overfitting. The ReLU activation function was used in all the FC layers of the DNN. The classification of the three classes is determined in the final FC layer through the utilization of the Softmax activation function.

### 3.3. The Structure of Our Proposed Model

**Figure 6:**
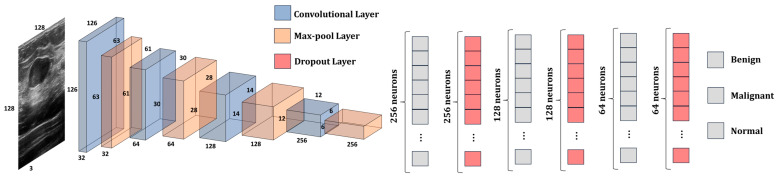
The architecture of our proposed model.

This subsection presents the architecture of our proposed model (Figure 6). The model is composed of two parts: the CNN, which is used for feature extraction, and the DNN, which is used to classify the features into one of the three classes: benign, malignant, or normal. CNN presents 4 convolutional layers and 4 max-pooling layers. The size of the kernels from the convolutional layers was chosen to be a 3 × 3 matrix, while the size of the kernels from the max-pooing layers is a 2 × 2 matrix. Additionally, the dimension of the input image was kept to 128 × 128 × 3 pixels. The first convolutional layer provides 32 feature maps; the second one has 64; the third one has 128; and the last one provides 256 feature maps. The activation function that was used by the model is Leaky ReLU. The model has 2,789,315 trainable parameters.

The number of trainable parameters for each convolutional layer and FC layer are computed using Equations (2) and (3), respectively.

(2) $$Conv\;Params=\left(W_{kernel}\;\cdot \;H_{kernel}+Kernels_{l}+1\right)\cdot \;Kernels_{l+1}$$

(3) $$FC\;Layer\;Params=\left(imput\;neurons+1\right)\cdot \;output\;neurons$$

*W_kernel_* and *H_kernel_* represent the width and height of the feature map matrix, while *Kernels_l_* and *Kernels_l+_*_1_ represent the number of feature maps from the current layer and from the next layer, respectively. To obtain a better understanding of the data passed through the model, Table 3 shows the shape of the tensors from each layer of the CNN and DNN, as well as the number of trainable parameters in each layer.

One can note that the number of trainable parameters (e.g., weights) increases as the number of convolutional layers increases, and starts decreasing starting from the second FC layer. Therefore, for the CNN, we chose to increase the number of feature maps to make sure that the network manages to learn and differentiate all the relevant information from the ultrasound image. For instance, the shape of the edge of the tumor is crucial in determining its nature, whether benign or malignant. Therefore, the CNN must have enough feature maps to be able to learn the information. On the other hand, the number of neurons in each hidden layer decreases from 256 to 3. This design decision was made to obtain an optimal number of trainable parameters and avoid introducing unnecessary computational costs without a significant increase in accuracy. For instance, if we had kept the number of neurons in each hidden layer to 256, the number of trainable parameters in the model would be 2,880,323, while the increase in performance would be negligible. Additionally, at every level, the network acquires new features by building upon the knowledge gained from the preceding layers. Therefore, by reducing the number of neurons, we aim to eliminate unnecessary information while preserving only the essential information of the image. 

The DNN is similar in structure to the one presented in Figure 5. The main differences between these two are that the batch normalization layer was removed, and the dropout rate was decreased by 10%. In this way, the value of the neurons that were set to 0 was decreased, and the data were better generalized.

Additionally, the proposed architecture was also validated on another similar dataset, having a structure similar to the dataset presented in Figure 3. The dataset is entitled Dataset B [39], and it was collected in the UDIAT Diagnostic Centre of the Parc Tauli Corporation, Sabadell, Spain. It contains 163 ultrasound images divided into two classes: benign (109 images) and malignant (54 images) breast tumors. The preprocessing steps are the same as the ones presented in Figure 2. Since the model was trained in three classes, that is, on benign, malignant, and normal classes, the number of output neurons was reduced from three to two. Additionally, the loss function was also changed from categorical cross-entropy to binary cross-entropy.

### 3.4. DL Frameworks

DL frameworks are very efficient as they provide a high-level programming interface for the training and validation of DL models. They are helpful when it comes to creating algorithms that contain complex operations, such as convolution or tensor operations, because they hide the complexity and provide intuitive functions. For the training and validation of the models, two popular Python-based frameworks were used: TensorFlow and Keras. Additionally, for data preprocessing, augmentation, and visualization, NumPy, OpenCV, and Matplotlib were used.

The Google Colab cloud-based platform was selected to conduct the experiments due to its solid hardware resources and served as a setting for developing and testing the proposed models. The Tesla T4 GPU utilized in this study is equipped with a total of 82 GB of GDDR6 memory. The current implementation involves the use of TensorFlow version 2.12.0 and Python 3.10.12.

## 4. Results

This section presents the experimental results obtained after training and validating both the state-of-the-art models using TL and our proposed architecture. Recall the data augmentation algorithm presented in Section 3. The experiments were conducted with two values for the multiplication factor, that is, 5 and 10, to observe and compare the impact of data augmentation on the training and validation processes. Therefore, the number of samples increased from 780 to 3900 for a multiplication factor of 5 and to 7800 for a multiplication factor of 10, respectively. During the training process, a monitoring technique called early stopping was introduced with a patience value of 4, that is, the number of epochs after which the training process is stopped if no accuracy or loss improvement is recorded. This approach mitigates overfitting by monitoring the validation loss and terminating the model if there is no improvement in the loss metric or if the model begins to overfit. Additionally, the batch size that was used to train the models was 32, with a learning rate of 10^−3^. What is more, each model was trained and evaluated on the test set five times, and the average value was taken into consideration ± the uncertainty value. Table 4 shows the results of the training process on the training, validation, and test sets for the multiplication factor of 5.

The best accuracy value on the test set was obtained by the DenseNet-121 model, with 95.63%, while the model that obtained the lowest accuracy value was ResNet-50. Additionally, the proposed architecture obtained competitive results compared to the other models, outperforming the ResNet-50, MobileNet-V2, Inception-V2, and Inception-V3 models.

To conduct this analysis, the following metrics were utilized: accuracy, precision, recall, F1-score, and specificity. Recall and specificity are essential parameters in medical image analysis as they have a direct impact on patient treatment decisions and healthcare expenditures. Ensuring sensitivity is of the utmost importance when it comes to early disease diagnosis and avoiding the occurrence of FNs. For instance, in the context of cancer screening, failing to detect a false positive result could lead to a delay in the patient’s treatment, perhaps exacerbating their condition. Ensuring a high level of specificity is crucial to prevent the need for unneeded testing, expenses, and invasive treatments. The mathematical expressions for the previously mentioned evaluation metrics are presented in Table 4. The compass tool serves as a means of comprehending the extent to which a model is inclined towards generating FPs or FNs.

The results after validating the models using the metrics are presented in Table 5 and the data augmentation algorithm with a multiplication factor of 5 is presented in Table 6. As per the type of cancer, the highest precision values for the benign, malignant, and normal classes were obtained by the VGG-16, DenseNet-121, and our proposed architecture, respectively. Additionally, the highest recall values were obtained for the benign, malignant, and normal classes by the DenseNet-121, InceptionResNet-V2, and VGG-16 models, respectively. The highest F1-Score was obtained by the DenseNet-121 and VGG-16 models for the normal and malignant classes. Finally, the highest specificity value was obtained by the VGG-16 model.

**Figure 7:**
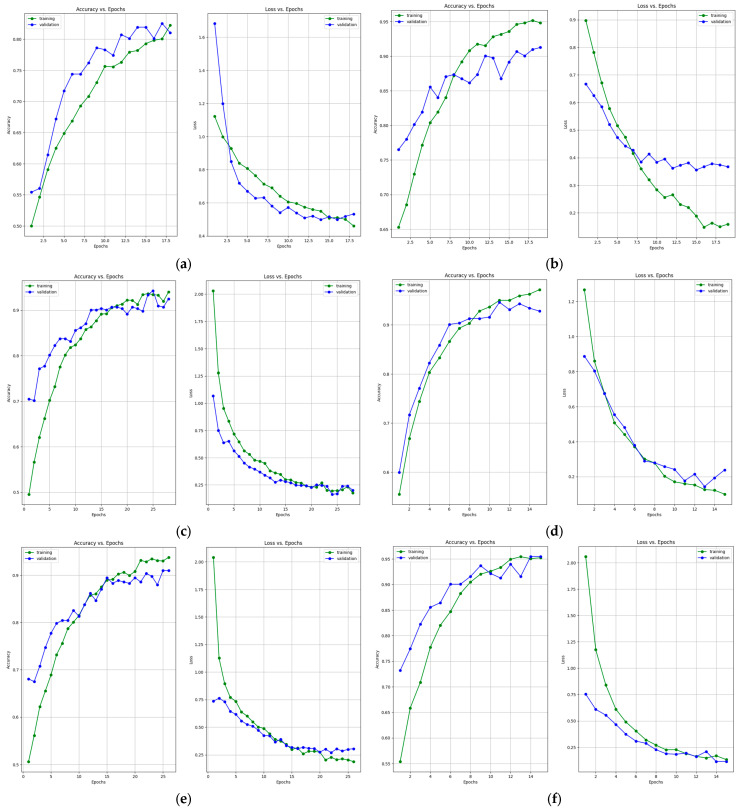
Accuracy and loss learning curves for (**a**) ResNet-50, (**b**) MobileNet-V2, (**c**) InceptionResNet-V2, (**d**) VGG-16, (**e**) Inception-V3, and (**f**) DenseNet-121 models after training with data augmentation with a multiplication factor of 5 on the BUS Images dataset.

**Figure 8:**
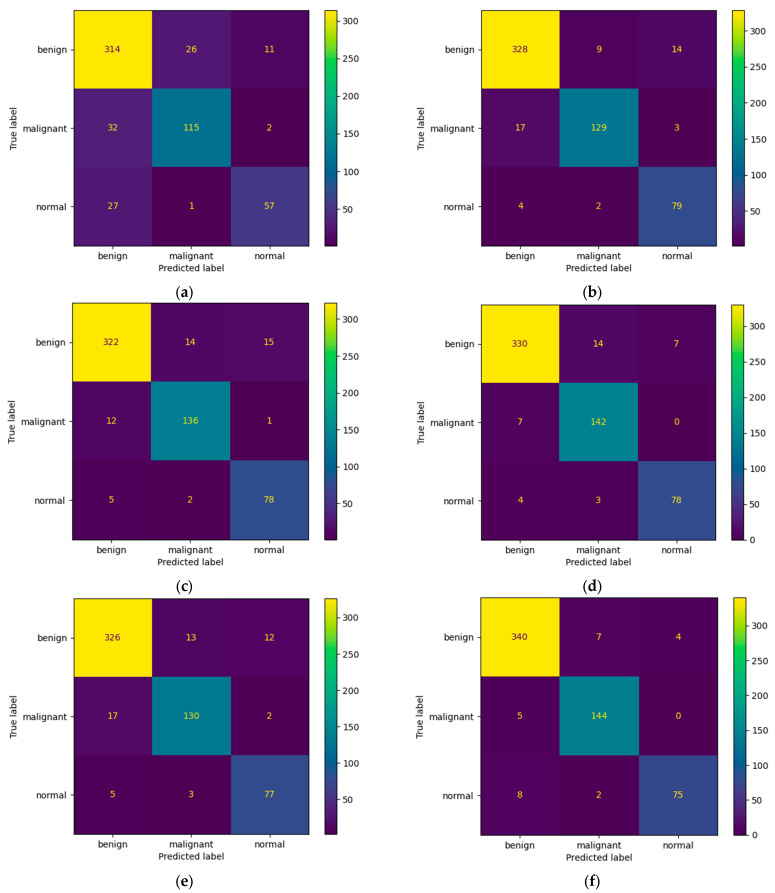
Confusion Matrices for (**a**) ResNet-50, (**b**) MobileNet-V2, (**c**) InceptionResNet-V2, (**d**) VGG-16, (**e**) Inception-V3, and (**f**) DenseNet-121 models after training with data augmentation with a multiplication factor of 5 on the BUS Images dataset.

Figure 7 presents the learning curves that resulted after training the ResNet-50, MobileNet-V2, InceptionResNet-V2, VGG-16, Inception-V3, and DenseNet-121 models. The models were trained for 26 epochs. However, the early stop optimization technique was applied to the ResNet-50, MobileNet-V2, VGG-16, and DenseNet-121 models. In Figure 8, the confusion matrices that were generated after training the data with the data augmentation algorithm with a multiplication factor of 5 are shown.

Regarding the number of TP predictions per class, the DenseNet-121 model achieved the highest value, that is, 340 for the benign class and 144 for the malignant class, while for the normal class, the MobileNet-V2 achieved the highest value, that is, 79.

**Figure 9:**
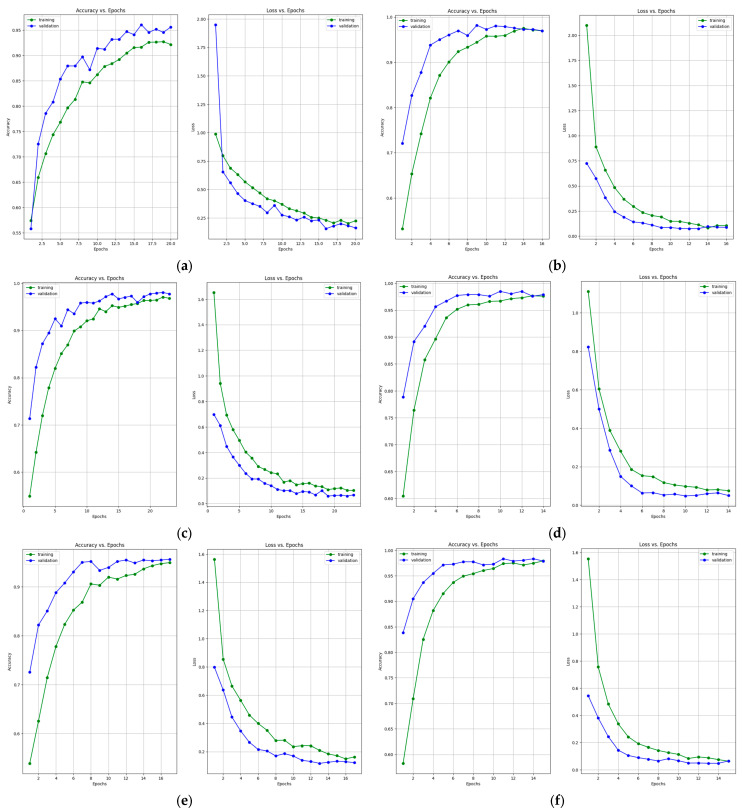
Accuracy and loss learning curves for (**a**) ResNet-50, (**b**) MobileNet-V2, (**c**) InceptionResNet-V2, (**d**) VGG-16, (**e**) Inception-V3, and (**f**) DenseNet-121 models after training with data augmentation with a multiplication factor of 10 on the BUS Images dataset.

**Figure 10:**
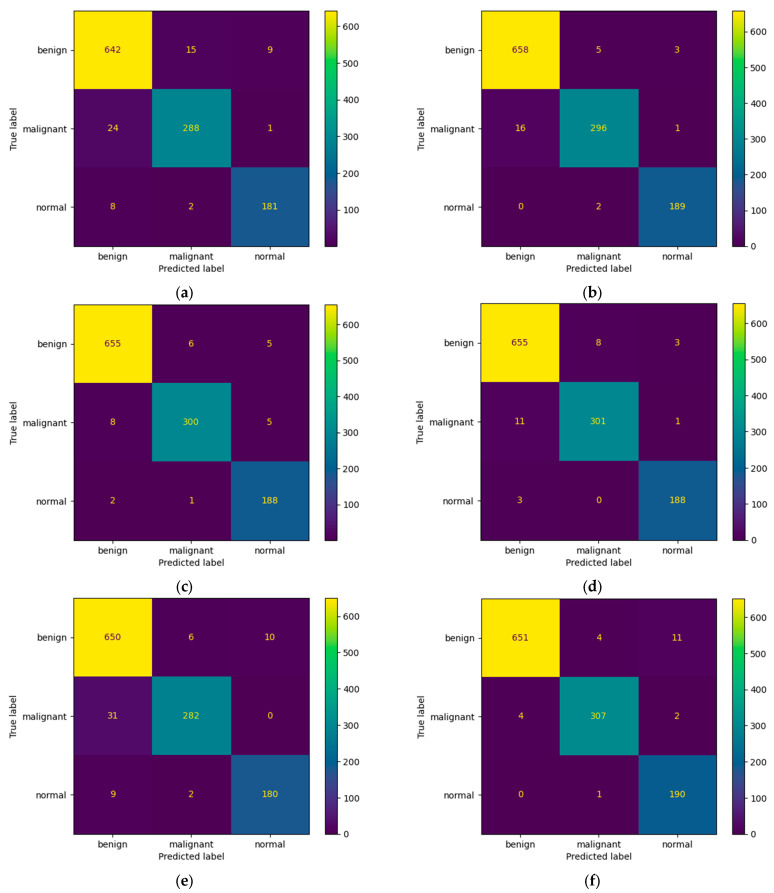
Confusion matrices for (**a**) ResNet-50, (**b**) MobileNet-V2, (**c**) InceptionResNet-V2, (**d**) VGG-16, (**e**) Inception-V3, and (**f**) DenseNet-121 models after training with data augmentation with a multiplication factor of 10 on the BUS Images dataset.

Table 7 and Table 8 present the accuracy report as well as the Precision, Recall, F1-Score and Specificity values after training the models using the data augmentation algorithm with a multiplication factor of 10. It can be clearly observed that, in this case, the highest accuracy on the test set was obtained by the DenseNet-121 model while the lowest accuracy was obtained by the ResNet-50 model. Additionally, the highest Precision values for the benign, normal, and malignant classes were obtained by the VGG-16 and our proposed architecture, respectively. Regarding the recall, the highest values were obtained by VGG-16 and MobileNet-V2 for the benign, malignant, and normal classes. The highest F1-Score was obtained by the following: InceptionResNet-V2 and VGG-16 for the benign class, DenseNet-121 and our proposed architecture for the malignant class and MobileNet-V2 for the normal class. Figure 9 and Figure 10 present the learning curves and confusion matrices after training the models using data augmentation with a multiplication factor of 10.

**Figure 11:**
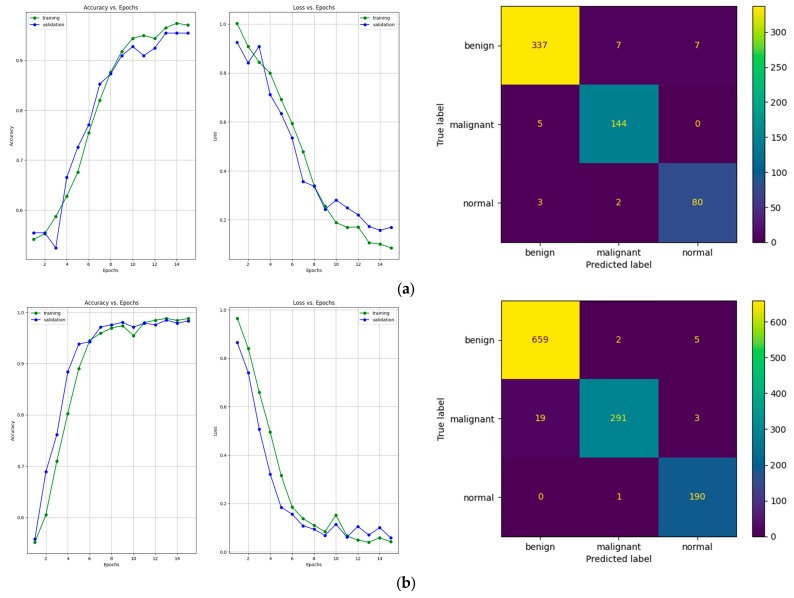
Learning curves and confusion matrices for our proposed architecture after training with the data augmentation algorithm with (**a**) a multiplication factor of 5 and (**b**) a multiplication factor of 10 on the BUS Images dataset.

Regarding the number of TP predictions per class, in this case, the MobileNet-V2 model achieved the highest value for the benign class, that is, 658 correct predictions. For the malignant and normal classes, the DenseNet-121 model achieved the highest results, that is, 307 and 190 correct predictions, respectively. Figure 11 shows a comparative overview of the learning curves and confusion matrices obtained after training our proposed architecture using data augmentation with a multiplication factor of 5 and 10, respectively, on the BUS Images dataset.

After training the model using the data augmentation algorithm with a multiplication factor of 5, the number of TP values was 337, 144, and 80 for the benign, malignant, and normal classes, respectively. After training the model using the data augmentation algorithm with a multiplication factor of 10, the number of TP values that were obtained was 659, 291 and 190 for the benign, malignant, and normal classes, respectively.

**Figure 12:**
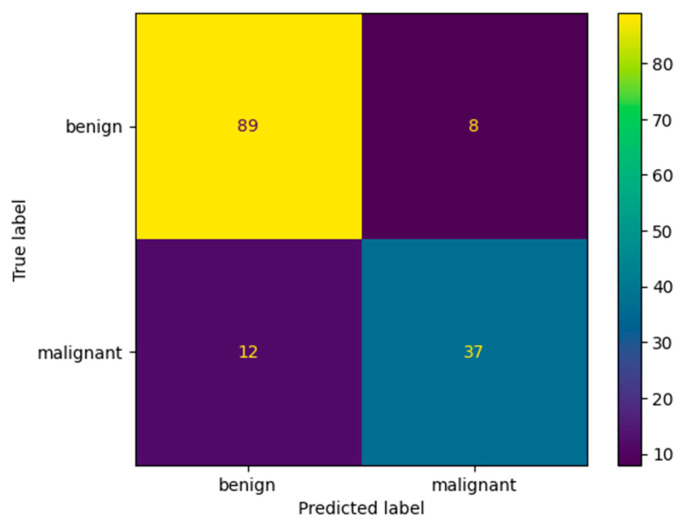
Confusion matrix for our proposed architecture after validation on Dataset B.

Figure 12 and Table 9 show the confusion matrix and performance results obtained after evaluating our model that was trained using the multiplication factor of 10 on Dataset B. The performance of the model on this dataset is a bit lower than on the BUS Images dataset. The accuracy obtained by the model is only 86.3% on Dataset B, compared to 96.75% on the BUS Images dataset. This could be because the augmentation factor that was used is quite high. Therefore, the model seems to overfit a bit on the BUS Images Dataset. However, the value of the accuracy is still higher than the one obtained by the ResNet-50 model after training it using the multiplication factor of 5, which is 84.01 ± 0.94.

In the light of the results, data augmentation proved to be a useful way of generating ultrasound images. One of the greatest advantages of this technique is that it leads to improved generalization. By exposing the model to a broader spectrum of input data variations throughout the training process, it acquires the ability to be more resilient and less responsive to minor fluctuations in the test data. In fact, the mean test accuracy obtained by all the models was 91.73% when using a data multiplication factor of 5, while the obtained test accuracy when using a data multiplication factor of 10 was 96.73%. Therefore, by increasing the amount of data, one can improve the overall performance of the models by 5% on the BUS Dataset.

## 5. Discussions

This section presents some observations and discussions based on the previously presented experimental results. The amount of data that is used to train the models drastically affects their performance. Therefore, the models that were trained using a factor of 10 in the data augmentation algorithm obtained better results compared to those using a factor of 5. In fact, the average accuracy that was obtained on the test dataset increased by 10.85%, 6.33%, 5.47%, 3.45%, 4.22%, 2.77%, and 2.91% for ResNet-50, MobileNet-V2, InceptionResNet-V2, VGG-16, Inception-V3, DenseNet-121, and our proposed architecture, respectively. Additionally, the learning curves presented in Figure 8 generalize the solution better due to the increase in the number of images from 3900 to 7800 samples. Even though the data augmentation increased the performance of the models, continuing to increase the multiplication factor could lead to overfitting. That is because excessive augmentation might introduce unrealistic variations that do not reflect the true distribution of the data, potentially harming generalization. Therefore, if the dataset is sufficiently diverse, the benefit of data augmentation in reducing overfitting might be limited.

Even though the state-of-the-art models obtained good results in terms of their overall accuracy, precision, recall, F1-score, and specificity, our proposed architecture outperformed some of them. According to Table 4, our proposed architecture outperformed the ResNet-14, MobileNet-V2, InceptionResNet-V2, and Inception-V3 models after testing them on the BUS Images dataset. Additionally, according to Table 7, our architecture outperformed the ResNet-50 and Inception-V3 models. Regarding the number of TP predictions that were obtained after training the models using the data augmentation algorithm with a multiplication factor of 5, our proposed architecture outperformed all the other models except DenseNet-121 on the benign and malignant classes, while on the normal class, it outperformed all the state-of-the-art models. Additionally, after using the data augmentation algorithm with a multiplication factor of 10, our proposed architecture outperformed all the other models on the benign class and outperformed the ResNet-50 and Inception-V3 models on the malignant class. In the benign class, it obtained the same number of TP values as DenseNet-121, which outperformed the other models. Even though our proposed architecture outperformed most of the state-of-the-art models after testing it on the BUS Images dataset, its performance dropped after validating it on Dataset B due to overfitting. However, it still outperforms the ResNet-50 model in terms of overall accuracy.

The architecture of our model was inspired by the VGG-16 model. However, instead of connecting two or three convolutional layers with one pooling layer, we used four pairs of convolutional and max pool layers for the Convolutional Neural Network. For the Deep Neural Network, we used three pairs of fully connected and dropout layers, unlike using only fully connected layers. Additionally, instead of using the ReLU activation function like VGG-16, we used Leaky ReLU [40], which has the advantage of addressing the dying ReLU problem by introducing a small negative slope for negative inputs, preventing neurons from becoming totally inactive.

The reason behind calculating the specificity value over all the samples rather than calculating it per class is due to imbalances in the data. In scenarios characterized by substantial class imbalance, the calculation of specificity for each class may be more influenced by the prevailing class, which, in this case, is the benign class with 437 samples. By aggregating specificity across all classes, we can ensure that the performance is equally evaluated for all classes, resulting in a more balanced assessment.

Apart from the classical performance metrics that can indicate the quality of the predictions a DL model is able to make, the training time also indicates the model’s capability to efficiently utilize the resources offered by the GPU. Therefore, Table 10 shows the average training time when using the data augmentation algorithm with a multiplication factor of 5 and 10, respectively.

Since the architecture of our proposed model is only composed of four convolution layers and four max pooling layers, the training time is drastically decreased compared to the other models, which are deeper in terms of the convolution and max pool layers. Therefore, in both cases, our model outperformed all the other models except MobileNet-V2 and Inception-V3.

Even though the experimental results demonstrated that the models could classify the type of breast tissue with relatively high accuracy, some limitations stand out. The limitations are similar to those mentioned by Yang L. et al. [41]. Firstly, the patients from whom the medical data were collected were from two geographical regions, that is, Baheya Hospital for Early Detection and Treatment of Women’s Cancer, Cairo, Egypt, and UDIAT Diagnostic Centre of the Parc Tauli Corporation, Sabadell, Spain. Therefore, whether BC evolves differently in other geographical regions and ethnic groups needs to be further investigated. Secondly, the models were only tested on two datasets; therefore, their performance on other medical data needs to be further investigated. Additionally, the models presented in this study can differentiate only between malign, benign, and healthy breast tissue. They do not differentiate between different subtypes of malignant and benign breast tissues. Lastly, the dataset does not mention whether the participants were exposed to risk factors such as faster menstruation, infertility, or late menopause, which directly impact the shape and evolution of BC [42].

## 6. Conclusions

This paper addresses the problem of the multi-instance classification of breast masses from ultrasound images. A set of six pre-trained architectures were employed as feature extractors, with their classification layers fine-tuned to align with the dataset and desired output. A data augmentation algorithm that multiplies the initial number of samples in the dataset by a factor of 5 and 10, respectively, is also introduced. Additionally, the architecture of a custom model is also presented and trained from the ground up and validated on two datasets, the BUS Images dataset and Dataset B. The result of this study is a comparison of the efficiency of the pre-trained models and our model in conjunction with the data augmentation algorithm applied to BC classification. The experimental results show that while the pre-trained models obtained good performance when detecting BC in the ultrasound images, our model outperformed some architectures such as ResNet-50, MobileNet-V2, Inception-V2, and Inception-V3 on the BUS Images dataset, obtaining 96.75% accuracy. However, on Dataset B, our model obtained a lower accuracy, that is, 86.3%. Additionally, the amount of time needed for the models to train was also considered and compared. Due to its lightweight structure, our model was able to train faster than some of the other pre-trained models, such as ResNet-50, VGG-16, InceptionResNet-V2, or DenseNet-121.

Further research directions are represented by first creating an end-to-end solution for BUS image detection and classification that can be integrated into the ultrasound scanner and adopted by other hospitals. Secondly, training the models using BUS images from other geographical regions can also be taken into consideration to increase their performance. Finally, federated learning could also be taken into consideration as a decentralized approach to secure the medical data obtained from the patients.

## Figures and Tables

**Table 1:** Summarized overview with related results.

Paper	Classification Type	Model	Accuracy [%]	Precision [%]	Recall [%]	F1-Score [%]	Sensitivity [%]	Specificity [%]
[11]	Binary	ResNet	99.7	99.59	-	-	97.53	97.8
		Inception-v3	97.66	97.64	-	-	97.64	97.59
		ShuffleNet	96.94	96.85	-	-	96.7	96.85
	Multi Class	ResNet	97.81	97.65	-	-	97.65	97.31
		Inception-v3	96.07	96.05	-	-	96.03	96
		ShuffleNet	95.79	95.7	-	-	95.7	95.5
[13]	Multi-Class	Xception	90.22	90.99	89.87	89.97	-	-
		DenseNet-201	90.22	91.18	97.22	88.95	-	-
		InceptionResNet-v2	85.87	86.86	81.31	83.6	-	-
		VGG-19	76.63	73.82	65.42	66.81	-	-
		ResNet-152	48.37	11.94	19.52	14.71	-	-
[14]	Multi-Class	MobileNet-v2	82.54	-	-	-	-	-
		Inception -v3	83.84	-	-	-	-	-
[15]	Multi-Class	VGG-19	93.76	88.11	-	87.84	87.72	94.97
		YOLO-v3	96.31	93.36	-	92.99	92.63	96.71
[16]	Multi-Class	VGG-16	81.11	-	-	80.05	-	-
		ResNet-50	85.4	-	-	93.51	-	-
		ResNeXt-50	85.83	-	-	80.53	-	-
[17]	Multi-Class	U-NET and SVM	94.4	96.2	86.7	91.2	-	-

**Table 2:** Summarized overview with the datasets used in the previously presented papers.

Paper	Datasets
[10]	BreakHis
[11]	BreakHis
[12]	693 BUS Images
[13]	BUS Images dataset and Private Dataset
[14]	BUS Images
[15]	170 Infrared images

**Table 3:** The shape of the feature maps from each layer of the proposed model.

Layer	Input Shape	Output Shape	Trainable Parameters
Input Layer	(128, 128, 3)	(128, 128, 32)	0
1st Convolution	(128, 128, 32)	(126, 126, 32)	896
1st Max-Pooling	(126, 126, 32)	(63, 63, 32)	0
2nd Convolution	(63, 63, 32)	(61, 61, 64)	18,496
2nd Max-Pooling	(61, 61, 64)	(30, 30, 64)	0
3rd Convolution	(30, 30, 64)	(28, 28, 128)	73,856
3rd Max-Pooling	(28, 28, 128)	(14, 14, 128)	0
4th Convolution	(14, 14, 128)	(12, 12, 256)	295,168
4th Max-Pooling	(12, 12, 256)	(6, 6, 256)	0
Flatten	(6, 6, 256)	(9216)	0
1st FC Layer	(9216)	(256)	2,359,552
1st Dropout	(256)	(256)	0
2nd FC Layer	(256)	(128)	32,896
2nd Dropout	(128)	(128)	0
3rd FC Layer	(128)	(64)	8256
3rd Dropout	(64)	(64)	0
4th FC Layer	(64)	(3)	195

**Table 4:** Training, validation, and test accuracy values obtained after training the models using TL and data augmentation with a multiplication factor of 5 on the BUS Images dataset.

Model	Training Accuracy [%]	Validation Accuracy [%]	Test Accuracy [%]
ResNet-50	89.5 ± 3.62	85.08 ± 3.16	84.01 ± 0.94
MobileNet-V2	98.27 ± 0.42	89.03 ± 0.15	91.36 ± 0.25
InceptionResNet-V2	98.05 ± 0.25	92.61 ± 0.75	92.22 ± 0.60
VGG-16	99.12 ± 0.17	93.97 ± 1.20	94.26 ± 0.25
Inception-V3	98.72 ± 0.5	89.90 ± 0.45	90.85 ± 0.26
DenseNet-121	99.09 ± 0.32	95.48 ± 0.87	95.63 ± 0.08
Our Architecture	98.55 ± 0.79	92.46 ± 0.37	93.84 ± 0.35

**Table 5:** Definition and mathematical formulas of the performance indicators.

Metric	Formula
Accuracy	$\frac{TP\;+\;TN}{TP\;+\;TN\;+\;FP\;+\;FN}$
Precision	$\frac{TP}{TP\;+\;FP}$
Recall	$\frac{TP}{TP\;+\;FN}$
F1-Score	$\frac{2\cdot TP}{2\cdot TP\;+\;FP\;+\;FN}$
Specificity	$\frac{TN}{TN\;+\;FP}$

**Table 6:** Precision, Recall, F1-Score and Specificity values after training the models using data augmentation with a multiplication factor of 5 on the BUS Images dataset.

Model	Tissue Type	Precision [%]	Recall [%]	F1-Score [%]	Specificity [%]
ResNet-50	Benign	88.5 ± 4.50	87 ± 2	88 ± 1	89.54 ± 1.17
	Malignant	81 ± 0.91	80 ± 3	80.5 ± 1.5	
	Normal	73.5 ± 7.25	76.5 ± 0.95	74.5 ± 0.5	
MobileNet-V2	Benign	94 ± 0.4	92.5 ± 0.5	93.5 ± 0.5	94.61 ± 1.72
	Malignant	89 ± 3	87 ± 0.2	87.5 ± 1.5	
	Normal	83.5 ± 1.5	92 ± 1.1	87 ± 0.3	
InceptionResNet-V2	Benign	96.5 ± 1.5	91 ± 1.1	93 ± 0.4	95.82 ± 0.96
	Malignant	89 ± 0.7	92 ± 1.2	90.5 ± 0.5	
	Normal	79 ± 4	93 ± 1.05	85. ± 1.5	
VGG-16	Benign	97.5 ± 0.5	94 ± 0.3	95.5 ± 0.5	97.59 ± 0.83
	Malignant	91.5 ± 2.5	95 ± 0.6	95 ± 0.3	
	Normal	89.5 ± 2.5	95.5 ± 3.5	92. 8 ± 1.2	
Inception-V3	Benign	93.3 ± 1.1	94.5 ± 1.5	93.5 ± 0.5	94.35 ± 1.47
	Malignant	91 ± 2.7	86.5 ± 0.5	88.5 ± 0.6	
	Normal	88 ± 3.2	88.5 ± 2.5	87.5 ± 0.5	
DenseNet-121	Benign	96 ± 0.2	97 ± 0.8	97 ± 0.3	97.28 ± 1.51
	Malignant	95 ± 1.2	95 ± 0.2	94.5 ± 0.5	
	Normal	94 ± 1.1	92 ± 4.4	93 ± 2.1	
Our Architecture	Benign	95.42 ± 0.58	95.75 ± 0.78	95.38 ± 2.16	92.15 ± 1.05
	Malignant	91.12 ± 0.53	94.91 ± 1.76	92.49 ± 3.42	
	Normal	95.64 ± 0.48	87.12 ± 0.64	91.72 ± 0.31	

**Table 7:** Training, validation, and test accuracy values obtained after training the models using TL and data augmentation with a multiplication factor of 10 on the BUS Images dataset.

Model	Training Accuracy [%]	Validation Accuracy [%]	Test Accuracy [%]
ResNet-50	98.24 ± 0.41	96.07 ± 0.13	94.95 ± 0.64
MobileNet-V2	99.22 ± 0.11	97.88 ± 0.38	97.69 ± 0.52
InceptionResNet-V2	98.97 ± 0.39	97.13 ± 0.79	97.69 ± 0.13
VGG-16	99.01 ± 0.08	98.49 ± 0.19	97.77 ± 0.29
Inception-V3	98.15 ± 0.28	94.87 ± 0.62	95.07 ± 0.41
DenseNet-121	99.53 ± 0.05	97.88 ± 0.25	98.11 ± 0.10
Our Architecture	98.70 ± 0.18	97.13 ± 0.16	96.75 ± 0.26

**Table 8:** Precision, Recall, F1-Score and Specificity values after training the models using TL and data augmentation with a multiplication factor of 10 on the BUS Images dataset.

Model	Tissue Type	Precision [%]	Recall [%]	F1-Score [%]	Specificity [%]
ResNet-50	Benign	94.6 ± 0.4	95.4 ± 0.4	95.8 ± 2.8	96.88 ± 1.29
	Malignant	94.4 ± 0.4	93.6 ± 0.6	93.4 ± 0.4	
	Normal	94.8 ± 0.8	95.6 ± 2.6	94.2 ± 0.6	
MobileNet-V2	Benign	97.8 ± 0.2	98.6 ± 0.6	97.8 ± 0.2	98.32 ± 0.15
	Malignant	98.3 ± 0.5	95.7 ± 1.2	96.6 ± 0.6	
	Normal	97.8 ± 0.2	98.4 ± 0.6	98.8 ± 0.2	
InceptionResNet-V2	Benign	97.6 ± 0.4	97.2 ± 0.6	98.6 ± 0.6	98.55 ± 0.21
	Malignant	97.6 ± 0.6	95.8 ± 0.4	96.4 ± 0.2	
	Normal	95.8 ± 0.8	98.2 ± 0.6	97.5 ± 0.4	
VGG-16	Benign	98.6 ± 0.4	98.6 ± 0.2	98.6 ± 0.4	98.62 ± 0.13
	Malignant	97.2 ± 0.6	96.6 ± 1.2	96.6 ± 0.6	
	Normal	98.4 ± 0.4	98.2 ± 0.2	98.2 ± 0.2	
Inception-V3	Benign	94.8 ± 0.8	98.2 ± 0.2	96.4 ± 0.6	96.70 ± 1.57
	Malignant	97.2 ± 0.4	90.6 ± 1.2	94.5 ± 0.4	
	Normal	95.6 ± 0.8	94.8 ± 0.6	94.2 ± 0.4	
DenseNet-121	Benign	98.4 ± 0.4	98.2 ± 0.6	98.4 ± 0.2	98.09 ± 0.51
	Malignant	98.2 ± 0.6	97.8 ± 0.2	97.4 ± 0.6	
	Normal	94.8 ± 1.2	98.4 ± 0.2	98.6 ± 0.4	
Our Architecture	Benign	96.4 ± 0.4	97.4 ± 0.2	97.2 ± 0.6	97.85 ± 0.85
	Malignant	98.6 ± 0.6	95.6 ± 1.4	97.4 ± 0.8	
	Normal	96.2 ± 0.4	94.3 ± 0.7	95.3 ± 1.2	

**Table 9:** Accuracy, Precision, Recall, F1-Score and Specificity values after validating our proposed architecture on the Dataset B.

Tissue Type	Accuracy [%]	Precision [%]	Recall [%]	F1-Score [%]	Specificity [%]
Benign	86.3	92	88	90	82.22
Malignant	86.3	76	82	79	

**Table 10:** Average training time after training the models on 3900 and 7800 samples, respectively.

Model	Average Training Time for 3900 Samples [min]	Average Training Time for 7800 Samples [min]
ResNet-50	34.45	65.8
MobileNet-V2	5.18	14.05
InceptionResNet-V2	47.34	81.72
VGG-16	71.91	134.8
Inception-V3	14.27	27.63
DenseNet-121	23.96	41.35
Out Architecture	14.28	28.83

## Data Availability

The BUS Images dataset presented in this study is openly available on Kaggle [18] and published in [17]. Dataset B was obtained upon request from the authors of paper [39].

## Abbreviations

The following abbreviations are used in this manuscript:

BC	Breast Cancer
BUS	Breast Ultrasound
CNN	Convolutional Neural Network
DL	Deep Learning
DNN	Deep Neural Network
FC Layer	Fully Connected Layer
FN	False Negative
FP	False Positive
TL	Transfer Learning
TN	True Negative
TP	True Positive
